# Cytogenomic Characterization of Giant Ring or Rod Marker Chromosome in Four Cases of Well-Differentiated and Dedifferentiated Liposarcoma

**DOI:** 10.1155/2022/6341207

**Published:** 2022-04-12

**Authors:** Hongyan Chai, Fang Xu, Autumn DiAdamo, Brittany Grommisch, Huanzhi Mao, Peining Li

**Affiliations:** ^1^Department of Genetics, Yale University School of Medicine, New Haven 06520, CT, USA; ^2^Prevention Genetics, Marshfield 54449, WI, USA

## Abstract

Chromosome and array comparative genomic hybridization (aCGH) analyses were performed on two cases of well-differentiated liposarcoma (WDLPS) and two cases of dedifferentiated liposarcoma (DDLPS). The results revealed the characteristic giant ring (GR) or giant rod marker (GRM) chromosomes in all four cases and amplification of numerous somatic copy number alterations (SCNAs) involving a core segment of 12q14.1q15 and other chromosomal regions in three cases. The levels of amplification for oncogenes OS9, CDK4, HMGA2, NUP107, MDM2, YEATS4, and FRS2 at the core segment or other SCNAs should be characterized to facilitate pathologic correlation and prognostic prediction. Further studies for the initial cellular crisis event affecting chromosome intermingling regions for cell-type specific gene regulation may reveal the underlying mutagenesis mechanism for GR and GRM in WDLPS and DDLPS.

## 1. Introduction

Liposarcomas (LPS) are the most common soft tissue sarcomas accounting for 20% of all sarcomas in adults. They originated from primitive mesenchymal cells and are found most often in extremities, particularly the thigh and retroperitoneum. According to the World Health Organization classification of soft tissue tumors, LPS are classified into atypical lipomatous tumors or well-differentiated LPS (ALT/WDLPS), dedifferentiated LPS (DDLPS), myxoid LPS, and pleomorphic LPS [1]. WDLPS/DDLPS accounts for 40–45% of all LPS. WDLPS is composed of locally aggressive mature adipocytes with low-grade malignancy; about 10% of WDLPS dedifferentiates to DDLPS with transformed nonlipogenic sarcomatous component and confers metastatic potential [2, 3].

Earlier cytogenetic analysis of WDLPS and DDLPS revealed characteristic clonal abnormalities by the presence of supernumerary giant ring (GR) and giant rod marker (GRM) chromosomes [4]. Cumulative data generated from fluorescence in situ hybridization (FISH) and comparative genomic hybridization (CGH) studies had shown that GR and GRM contain amplified materials from the 12q13q21 region and other variable chromosomal regions [5–8]. Further analysis using array CGH (aCGH) defined somatic copy number alterations (SCNAs) in the GR and GRM. Several studies revealed discontinuous amplified SCNAs and gene contents from a core region at 12q13q15 juxtaposed to other regions of 1p21p32, 1q21q24.4, 6q23q24, and 13q32.1q32.3 [9–16]. Recurrent SCNAs and genes of importance in the oncogenesis pathway, tumor classification, and prognostic value had been indicated, but the guidelines in analyzing and reporting cytogenomic findings for LPS are still lacking. In the present study, we performed chromosome and aCGH analyses on one case of WDLPS and two cases of DDLPS. These results further demonstrated the spectrum of SCNAs and provided reference for diagnostic interpretation and insight for the forming mechanisms of GR and GRM.

## 2. Case Presentation

### 2.1. Pathologic Findings for Four Cases

#### 2.1.1. Case 1

An 85-year-old woman initially presented with a painless mass in the right thigh/knee, and magnetic resonance imaging (MRI) revealed a 16 cm tumor. The patient underwent a resection and remained free of recurrence. The tumor showed yellow lobulated segment of adipose tissue (17 × 13 × 4 cm) with homogeneous lobulated yellow parenchyma on cut section. Microscopic exam found well-differentiated adipocytes with mild to moderate atypia. This tumor was classified as ALT/WDLPS.

#### 2.1.2. Case 2

A 71-year-old man presented with history of 9 × 4 cm retroperitoneal masses and a concordant 11 cm chest wall mass. The patient underwent resection, but tumor recurred in the retroperitoneum four months later. The patient again underwent resection with recurrence in the retroperitoneum two years later. The tumor showed well-encapsulated mass (9 × 6 × 2 cm) of gelatinous and lobulated parenchyma on cut section with attached fascia (9.5 × 4 × 1.1 cm). Microscopic exam found mature fat with areas of nuclear atypia increased from previous resection specimens and areas of sclerosis. This tumor was classified as sclerosing subtype of WDLPS.

#### 2.1.3. Case 3

An 85-year-old man presented with a retroperitoneal tumor. The patient underwent uneventful resection and was with absence of recurrent disease one year following therapy. The tumor showed encapsulated perinephric mass (16 × 11 × 9 cm) with fleshy, heterogeneous multilobulated on cut surface with focal areas of hemorrhage. Microscopic exam showed alternating areas of well-differentiated liposarcoma and pleomorphic spindle cell sarcoma positive for smooth muscle actin. Multiple resection margins were positive for tumor. This tumor was classified as high-grade DDLPS.

#### 2.1.4. Case 4

A 61-year-old man initially presented with right flank pain. MRI revealed a 16 cm retroperitoneal tumor. Right nephrectomy and tumor resection were performed with subsequent chemotherapy. The patient returned with recurrence two years later. The tumor with yellow multinodular mass (16 × 11.5 × 10.5 cm) was noted at the lower pole of the kidney. On cut section, the mass is yellow-tan and focally gelatinous with areas of hemorrhage and possible necrosis. Microscopic exam found spindle cells with low to moderate cellularity and increased mitotic index with 5–10% necrotic component. Tumor periphery shows well-differentiated adipose tissue with occasional pleomorphic atypical cells. This tumor was classified as DDLPS with low to intermediate grade dedifferentiation arising from a WDLPS.

### 2.2. Cytogenomic Results from Chromosome and aCGH Analyses

Cytogenetic analysis was performed on GTG banded metaphase chromosomes prepared from cultured tumor cells following the laboratory's standardized protocols; twenty metaphases were analyzed for each sample, and clonal abnormalities were karyotyped [17, 18]. aCGH analysis using Agilent SurePrint Human Genome Microarray 4 × 60 K kit and Agilent DNA Analytical (version 4.0) (Agilent Technologies Inc., Santa Clara, CA) to detect SCNAs on genomic DNAs extracted from cultured tumor cells was performed as previously described [19, 20]. Log2 ratio (L2R) from the tumor DNA over control DNA was used to measure the copy numbers of SCNAs. The SCNAs with level of amplification of four or more copies as defined by L2R > 2 are considered as amplicons. Benign copy number variants from the Database of Genomic Variants (https://projects.tcag.ca/variation/) were excluded. The base pair designation follows the March 2006 Assembly (NCBI36/hg18) on the UCSC Human Genome browser (https://genome.ucsc.edu/). The aCGH finding from each case was compared with the chromosomal abnormality to further define the breakpoint and the gene content involved. The size of the GR or GRM was estimated by the sum of amplified SCNAs times their copy numbers.

**Figure 1:**
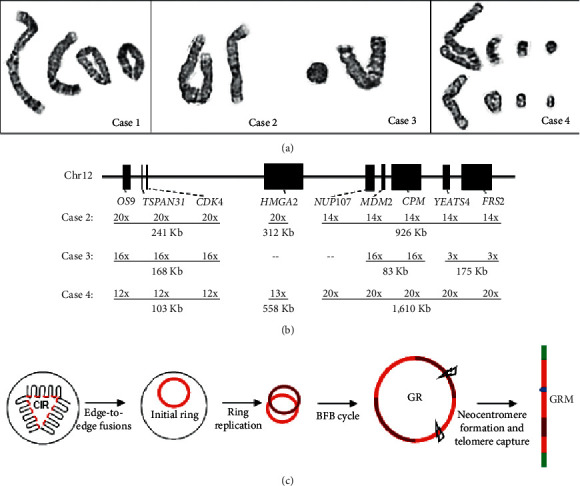
Cytogenomic findings in the three cases and a cellular process for the giant ring (GR) or giant rod marker (GRM). (a) Chromosome results showing GR or GRM in cases 1 to 4. (b) Amplification levels of SCNAs and putative oncogenes in the core segment of 12q14.1q15 in the three cases are given by number of copies and size of amplicons in Kb. Dash line “--” indicates normal two copies without amplification. (c) A diagram showing a cellular crisis in a chromosome intermingling region (CIR) for initial ring formation by an erroneous replication (red line), the breakage-fusion-bridge (BFB) cycles for an amplified GR, and neocentromere formation (blue dot) and telomere capture (green bar) for the stabilized GRM.

The age, gender, histopathologic findings, and chromosome results for the four cases are summarized in Table 1. Chromosomally detected GR or GRM in these four cases are shown in Figure 1(a). Case 1 had a small cell pellet and did not yield sufficient DNA for further analysis, while cases 2, 3, and 4 had sufficient DNA for aCGH analysis. In Case 2, aCGH detected chromosomal duplications in 5q and 19q, monosomy 13, and clusters of 54 amplified SCNAs located at 1q23.1q25.3, 6q21, 6q22.31, 6q24.2q24.3, 8q21.11q24.3, and 12q14.1q21.2 (Supplemental Figure 1). The cumulative size of these 54 SCNAs is about 35 Mb and the average size of an amplicon is approximately 652 Kb; the estimated size of GRM is 294 Mb, which indicated an eight-fold increase of all amplicons (Supplemental Table 1). The highest amplified SCNAs with 19 to 20 copies and their gene content were as follows: a 424 Kb amplicon at 1q23.1 (CD1A/C/B/D, OR10T2/K2/K1/R2, OR6Y1, OR10X1/Z1, and SPTA1 genes), a 115 Kb amplicon at 1q24.2 (SAC, BRP44, and IQWD1 genes), a 984 Kb amplicon at 6q24.3 (SAMD5 and SASH1 genes), a 241 Kb amplicon at 12q14.1 (OS9, CENTG1, TSPAN31, CDK4, MARCH9, CYP27B1, METTL1, FAM119B, TSFM, AVIL, CTDSP2, and XRCC6BP1 genes), a 312 Kb amplicon at 12q14.3 (HMGA2 gene), and a 143 Kb amplicon at 12q21.2 (SYT1 gene).

In Case 3, aCGH detected large deletions of 1p21.2p11.2 and 1q31.1q43, duplications of 4p13p16.3, 6q25.1q25.3, 9q13q34.3, and 17p13.3p11.2, trisomy 6, and clusters of 61 amplified SCNAs at 1q21.1q25.3, 5q33.3, 5q34q35.1, 11q23.1q25, 12p13.31p11.1, 12q13.11q15, 12q21.1q21.31, and 22q12.1 (Supplemental Figure 2). The cumulative size of these 61 SCNAs is about 31 Mb, and the average size of an amplicon is 514 Kb; the estimated size of the GRM is 222 Mb, which indicated a seven-fold increase of all amplicons (Supplemental Table 2). The highest amplified SCNAs with 16 to 20 copies and their gene content were: a 131 Kb amplicon at 1q42.12 (ITPKB), a 75 Kb amplicon at 11q24.3 (ETS1), a 193 Kb amplicon at 11q25 (OPCML), a 108 Kb amplicon at 12p11.23 (ITPR2), a 162 Kb amplicon at 12p11.22 (CCDC91), a 337 Kb amplicon at 12q13.11 (SLC38A1/2), a 2,690 Kb amplicon at 12q14.1 (OS9, CENTG1, TSPAN31, CDK4, MARCH9, CYP27B1, METTL1, FAM119B, TSFM, AVIL, and CTDSP2), a 67 Kb amplicon at 12q15 (RAP1B and LOC643752), and an 83 Kb amplicon at 12q15 (MDM2 and CPM).

In Case 4, aCGH detected duplications of 16q21q24.3 and 20q13.2q13.33, and clusters of 39 amplified SCNAs at 2q21.1q21.3, 2q36.2q36.3, 5p13.1p12, 8q12.1q21.11, 11q22.1q22.3, 12q13.13q23.1, 19q13.2, 19q13.42, and 22q13.31q13.32 (Supplemental Figure 3). The cumulative size of these 39 SCNAs is about 42 Mb, and the average size of an amplicon is 1,082 Kb; the size of the GRM is estimated as 338 Mb, which indicated an eight-fold increase of all amplicons (Supplemental Table 3). The highest amplified SCNAs with 20 copies, and their gene contents were as follows: a 1,610 Kb amplicon at 12q15 (NUP107, SLC35E3, MDM2, CPM, CPSF6, LYZ, YEATS4, FRS2, CCT2, LRRC10, BEST3, RAB3IP, and CNOT2), a 717 Kb amplicon at 12q15 (CPSF6, LYZ, YEATS4, FRS2, CCT2, LRRC10, BEST3, and RAB3IP), a 436 Kb amplicon at 12q21.33 (DUSP6, WDR51B, GALNT4, and ATP2B1), and an 863 Kb amplicon at 12q21.33 (C12orf12, EPYC, KERA, LUM, and DCN).

Shared amplified SCNAs in all three cases were at 12q14.1 and 12q15 and in two cases were at 1q23.1, 1q24.2, 1q24.3, 1q25.1.6q24.3, 8q12.1, 8q21.11, 12q14.1, 12q14.3, 12q15, and 16q22.1. The shared amplified SCNAs at 12q14.1 contain the OS9, CENTG1, TSPAN31, CDK4, MARCH9, CYP27B1, METTL1, FAM119B, TSFM, AVIL, CTDSP2, and XRCC6BP1 genes, and the SCNAs at 12q15 contain the MDM2, CPM, and FRS2 genes.

## 3. Discussion

Recurrent and discontinuous SCNAs in the form of GR and GRM are the unique genomic aberrations for WDLPS and DDLPS. High-level amplifications of SCNAs at 12q14.1q15 were noted in almost all cases with GR and GRM [10–16]. Structural analysis on the genomic architecture of GR and GRM revealed that a 12 Mb core segment of 12q14.1q15 is pivotal to the initial formation of the episomal ring structure, and transcriptional analysis confirmed overexpression of amplified genes OS9 and CDK4 at 12q14.1, HMGA2 at 12q14.3, and NUP107, MDM2, YEATS4 and FRS2 at 12q15 [21]. Results from previous studies and present cases indicated that the MDM2 gene probably involved in the initial event and was consistently amplified and overexpressed, therefore should be considered as the main driver gene. The MDM2 is essential for ubiquitination and degradation of the tumor suppressor TP53. MDM2 binds to negatively regulate TP53, preventing nuclear translocation and transcription, and promoting it degradation by E3 ubiquitin ligase. MDM2 amplification could result in reduced levels of TP53, which impairs the apoptotic activity and induces cellular proliferation [3]. It has been suggested that the amplification of oncogenes MDM2, CDK4, and YEATS4 and adipocytic differentiation factor HMGA2 drive tumor genesis [16]. The protein coded by the CDK4 gene affecting the CDKN2A/CDKN2B/CDK4/CCND1 pathway is thought pivotal in WDLPS and DDLPS oncogenesis [12]. Amplification of FRS2 gene may play a functional role in high-grade LPS through the activation of the FGFR/FRS2 signaling pathway [22, 23]. The amplification of HMGA2 was associated with ALT/WDLPS and a good prognosis, while amplification of CDK4 was associated with DDLPS and a poor prognosis [14]. The present Case 2 showed amplification of (OS9-CDK4)/HMGA2/(MDM2-YEATS4-FRS2), Case 3 showed amplification of (OS9-CDK4)/MDM2/(YEATS4-FRS2), and Case 4 showed amplification of (OS9-CDK4)/HMGA2/(MDM2-YEATS4-FRS2) (Figure 1(b)). These patterns supported the MDM2 as the main driver gene and other genes as codriver or passenger genes. The SCNAs in other chromosomes including amplifications of 1p32.2 (JUN), 12q13.3(GLI1), 6q23.3 (MAP3K5), losses of 6p, 6q, 11p, 11q, and 13q, and gains of 14q have been reported to be associated with DDLPS [10, 13, 15]. However, these SCNAs were not observed in the present three cases. The distinction of driver and passenger genes in the GR and GRM remained inconclusive. A description on the level of amplification of these oncogenes should be provided in the aCGH report to facilitate more precise correlation with histopathologic findings. Further integrated genetic and functional analyses of these putative candidate genes on the oncogenesis and progression in LPS could lead to better genotype-phenotype correlation for diagnostic cytogenomic interpretation [24–26].

Tens to hundreds of genomic rearrangements occur in a one-off cellular crisis for cancer development has been reported in a phenomenon termed chromothripsis [27]. Chromothripsis usually involved continuous crisscross back and forth segmental deletions and duplications within one chromosome or across several chromosomes. The GR and GRM showed massive genomic rearrangements involving mainly the amplifications of discontinuous SCNAs. The mechanism of formation involves initiation of a ring chromosome of the core segment, further amplification through a break-fusion-bridge (BFB) process, and final linearization through telomere capture [21]. This phenomenon could be similar but more complicated than constitutional ring chromosomes exhibiting segmental deletions and duplications during the repairing of DNA breakages and dynamic mosaicism through the BFB process during mitosis [28, 29]. The present cases showed the involvement of 39 to 61 SCNAs with an average size from 500–1,000 Kb and an average of eight-fold increase in amplification. Genomic sequencing analysis revealed edge-to-edge fusions to form the initial ring with the core segment and about eight to ten folds increase from the initial ring to the GRM. This observation suggested a one-off event of cellular crisis during the initiation stage followed by three to four cycles of BFB to the stabilized GR or GRM [21]. Recent studies on spatial organization and interactions of chromosome territories inside nucleus indicated the presence of chromosome intermingling regions (CIRs) serving as mechanical hotspots that harbor cell-type specific gene clusters for transcriptional machinery [30, 31]. Peculiar genomic regions from some chromosomes were looped out to an CIR for transcription; this closeness of certain genes has been found to be positively correlated with the occurrence of recurrent translocations in different types of tumors [32]. The recurrence of core segment and related chromosome regions in the initial ring for GR or GRM probably indicated the presence of cell-type specific CIR for gene expression regulation. It is hypothesized that a cellular crisis that delays the termination of an active transcriptional machinery followed by erroneous replication of the clusters of genes in the CIR could lead to the formation of an initial ring. Further amplification of this ring by BFB and overexpression of oncogenes cause delay in G1/S transition and selective growth advantage for tumor development [33] (Figure 1(c)). Current analysis revealed the complex genomic rearrangements of the stabilized GR or GRM. Studies of cases in the early stage of initial ring formation or on a cellular model under certain crisis condition are needed to validate this hypothesis.

Cytogenomic characterization on the level of amplification and the list of candidate oncogenes with pathogenic and prognostic significance from the core segments and other amplicons in the GR and GRM should be standardized in the cytogenomic analysis for WDLPS and DDLPS. Further studies on the initial event of ring chromosome formation might reveal molecular mechanism regulating cellular activities from transcription to replication. More reliable genotype-phenotype correlation for LPS tumor classification and prognostic prediction could provide guidance for diagnostic interpretation and treatment.

## Figures and Tables

**Table 1:** Pathologic and cytogenetic findings in four cases.

Case no.	Age (yr)	Sex	Subtype	Origin of sample	Location	Karyotype^*∗*^
1	85	F	ATL/WDLPS	Primary	Right thigh	47,XX,+gr [8]/47,idem,+10,-13,+grm [3]
2	71	M	WDLPS	Recurrent	Retroperitoneum	46,XY,-13,+grm [10]
3	85	M	DDLPS	Primary	Retroperitoneum	48,XY,del (1) (q31q43),t(13; 16) (q14; q22),+grm,+r [2]
4	61	M	DDLPS	Recurrent	Retroperitoneum	49–50,XY,+1-2grm,+3r [cp15]

^*∗*^gr, giant ring; grm, giant rod marker; r, ring.

## Data Availability

No data were used in this study.

## Supplementary Materials

Supplementary Materials Supplemental Tables 1–3 and Figures 1–3: genomic SCNAs detected in cases 1–3, respectively.
