# Supplementary material for: Cytogenomic Characterization of Giant Ring or Rod Marker Chromosome in Four Cases of Well-Differentiated and Dedifferentiated Liposarcoma
Source: Case Rep Genet. 2022 Apr 12;2022:6341207. doi: 10.1155/2022/6341207 (PMC9018199; doi:10.1155/2022/6341207)
Supplement: Supplementary Materials — Supplemental Tables 1–3 and Figures 1–3: genomic SCNAs detected in cases 1–3, respectively. [file 6341207.f1.zip › 6341207.f1/Sup Figs 1-3_V6.pdf]

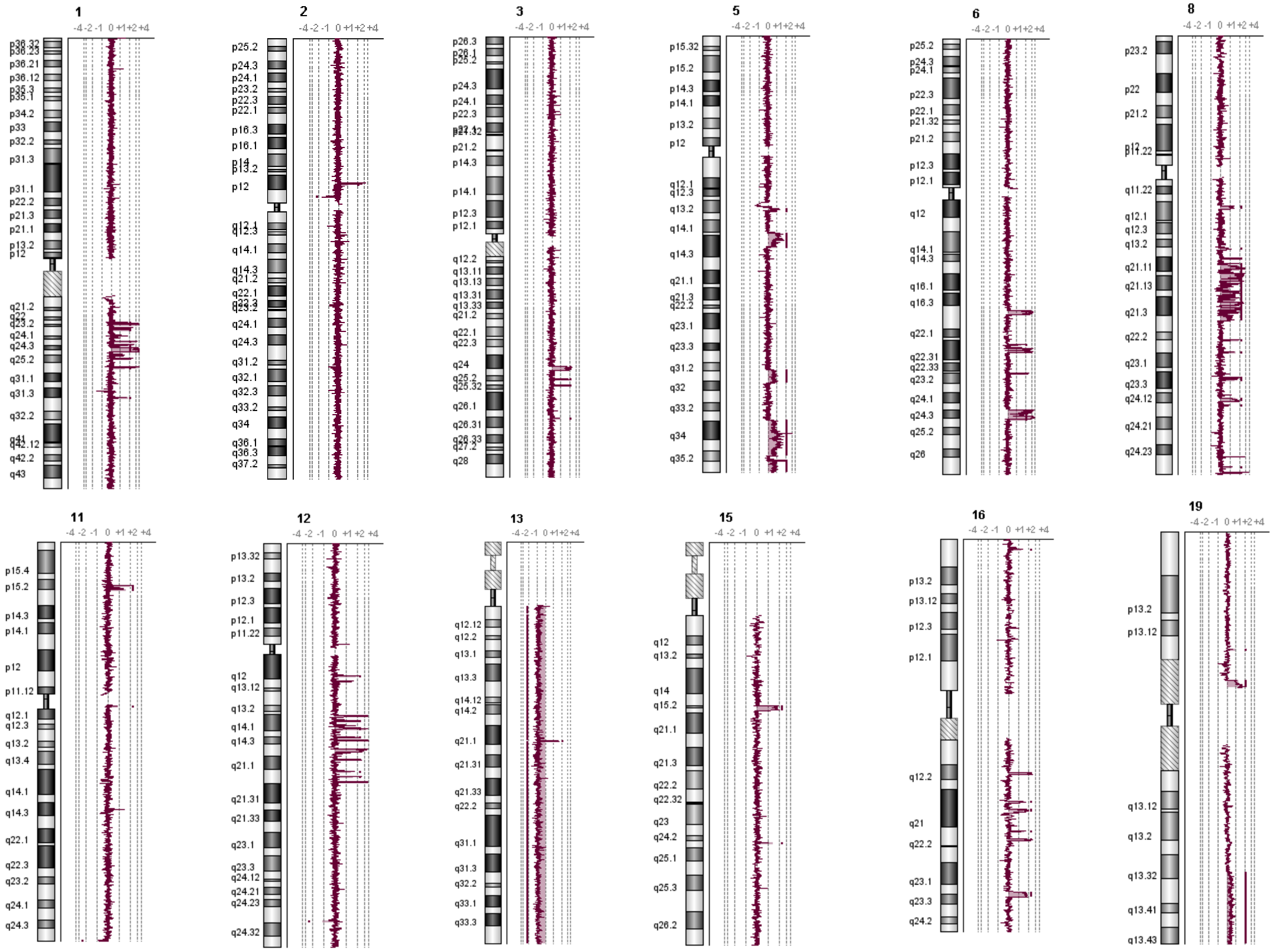

Supplemental Figure 1. Results of aCGH for case 2

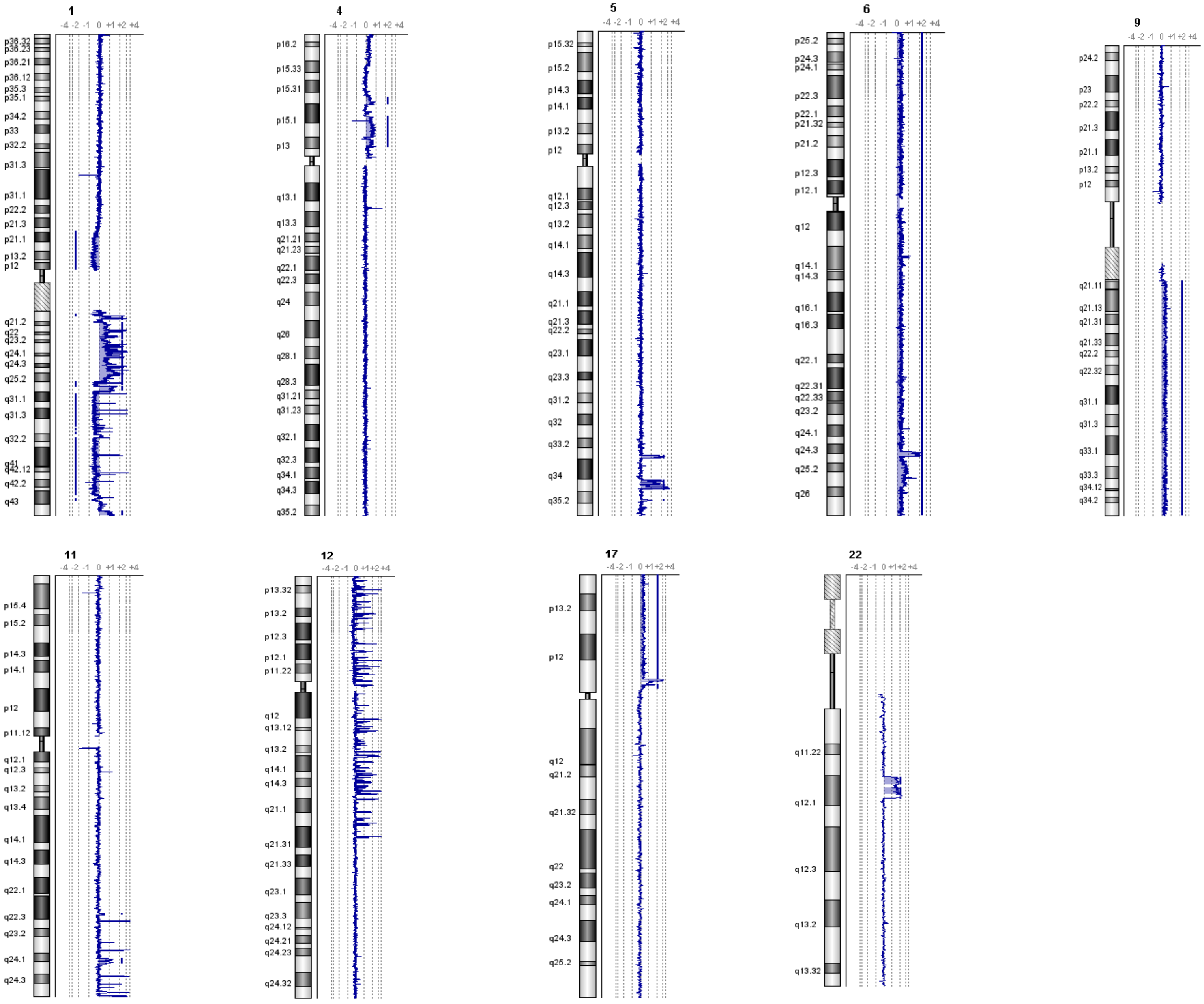

Supplemental Figure 2. Results of aCGH for case 3

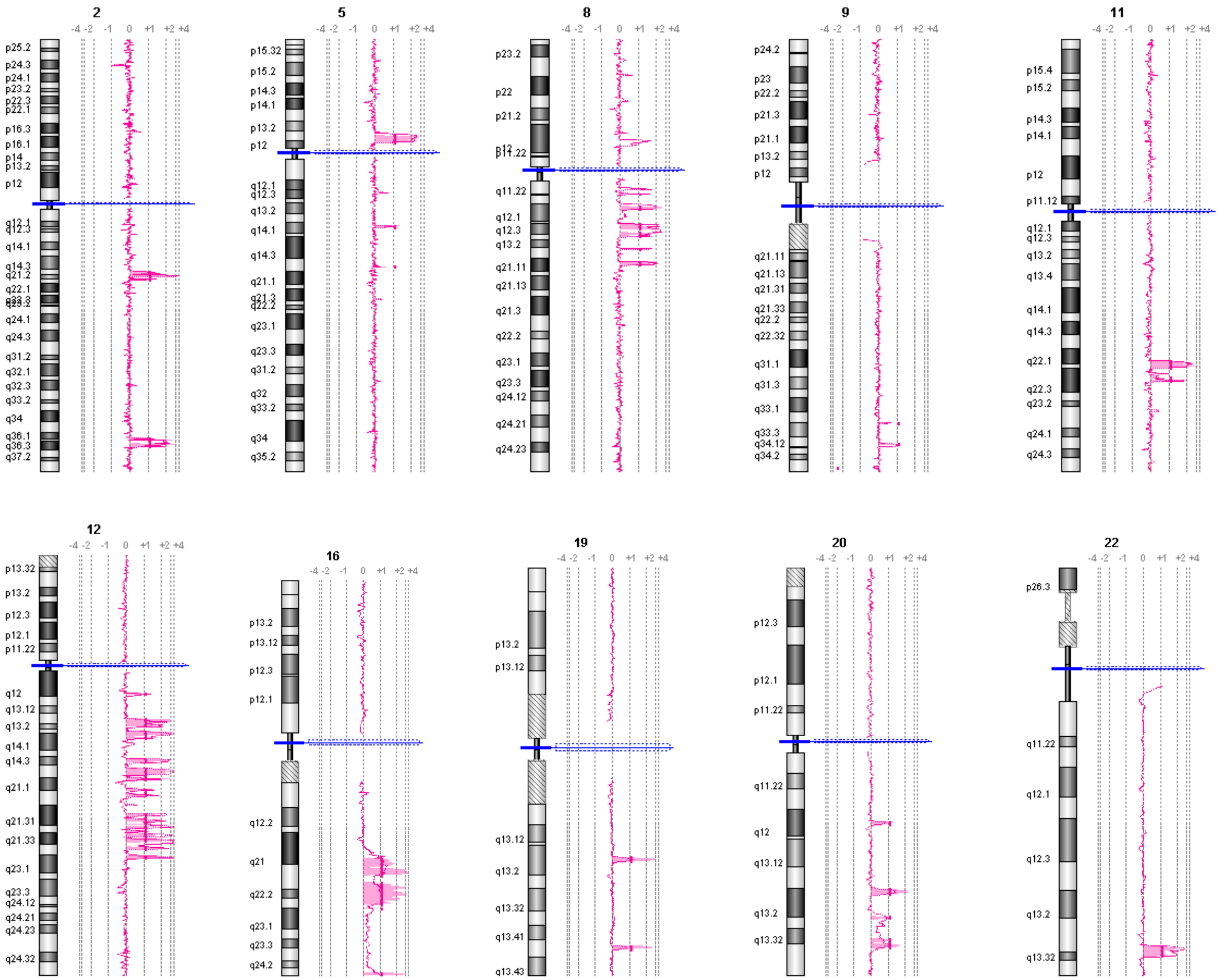

Supplemental Figure 3. Results of aCGH for case 4
